# Exploring the similarities between risk factors triggering depression in humans and elevated in-cage “inactive but awake” behavior in laboratory mice

**DOI:** 10.3389/fvets.2024.1348928

**Published:** 2024-03-28

**Authors:** Oceane Schmitt, Emily Finnegan, Anna Trevarthen, Chanakarn Wongsaengchan, Elizabeth S. Paul, Michael Mendl, Carole Fureix

**Affiliations:** Bristol Veterinary School, University of Bristol, Bristol, United Kingdom

**Keywords:** depression-like states, waking inactivity, maternal separation, environment, rodents

## Abstract

**Introduction:**

Depression is a human mental disorder that can also be inferred in non-human animals. This study explored whether time spent inactive but awake (“IBA”) in the home-cage in mice was further triggered by risk factors similar to those increasing vulnerability to depression in humans (early life stress, genetic predispositions, adulthood stress).

**Methods:**

Eighteen DBA/2 J and 18 C57BL/6 J females were tested, of which half underwent as pups a daily maternal separation on post-natal days 2–14 (early-life stress “ELS”) (other half left undisturbed). To assess the effect of the procedure, the time the dams from which the 18 subjects were born spent active in the nest (proxy for maternal behavior) was recorded on post-natal days 2, 6, 10 and 14 for 1 h before separation and following reunion (matched times for controls), using live instantaneous scan sampling (total: 96 scans/dam). For each ELS condition, about half of the pups were housed post-weaning (i.e., from 27 days old on average) in either barren (triggering IBA and depression-like symptoms) or larger, highly enriched cages (*n* = 4–5 per group). Time mice spent IBA post-weaning was observed blind to ELS treatment using live instantaneous scan sampling in two daily 90-min blocks, two days/week, for 6 weeks (total: 192 scans/mouse). Data were analyzed in R using generalized linear mixed models.

**Results:**

The dams were significantly more active in the nest over time (*p* = 0.016), however with no significant difference between strains (*p* = 0.18), ELS conditions (*p* = 0.20) and before/after separation (*p* = 0.83). As predicted, post-weaning barren cages triggered significantly more time spent IBA in mice than enriched cages (*p* < 0.0001). However, neither ELS (*p* = 0.4) nor strain (*p* = 0.84) significantly influenced time mice spent IBA, with no significant interaction with environmental condition (ELS × environment: *p* = 0.2861; strain × environment: *p* = 0.5713).

**Discussion:**

Our results therefore only partly support the hypothesis that greater time spent IBA in mice is triggered by risk factors for human depression. We discuss possible explanations for this and further research directions.

## 1 Introduction

Clinical depression^1^ is a mental disorder characterized by the co-existence of several debilitating symptoms (depressed mood, loss of pleasure or interest in activities, poor concentration, feelings of excessive guilt or low self-worth, hopelessness, suicidal thoughts, changes in sleep and appetite/weight, and/or fatigue) for at least two weeks (1, 2). Clinical depression affects about 5% of the adult human population worldwide (3) and has a higher prevalence in women (1–8). As stated by the diathesis-stress model (a common framework modelling the etiology of complex mental disorders), individuals made vulnerable by genetic predisposition and/or exposure to early-life stressors (e.g., childhood neglect and abuse) are more prone to develop the symptoms of depression when subjected to stressors later in life, compared to individuals not exposed to these risks factors (9, 10).

Like humans, captive and domestic animals can be exposed to these risk factors, i.e., early life stress [e.g., mother-infant early separation, e.g., (11, 12)], their stress-mediated responses can be influenced by genetic factors [e.g., (13, 14)], and they can be exposed to stress during adulthood. The latter includes exposure to a variety of short-, mid- and longer-term environmental stressors (e.g., confinement, unoptimized feeding regimes), social stressors (e.g., mixing, crowding, social restrictions), and pain due to husbandry practices (e.g., disbudding, castration) or health issues (e.g., keel bone damage, mastitis, lameness) [reviewed in Lecorps et al. (15) and Appleby et al. (16)]. Among the animals’ reactions to such impoverished/chronically stressful environments are elevated levels of waking inactivity, i.e., being inactive but awake “IBA” (motionless, eyes open), in the home environment [reviewed in Fureix and Meagher (17) and MacLellan et al. (18)]. Increased inactivity is a feature of human clinical depression (2). We therefore propose that, in animals exposed to chronically barren/restricted environments, greater levels of IBA could indicate depression-like states (19); and that, like in humans, the vulnerability to develop these states are increased by genetic and early life risk factors.

In mice (*Mus musculus*), elevated IBA was first mentioned as a “pathological form of behavior” and “posture of depression” 30 years ago, in a social defeat study where half of the defeated mice displayed “nose in the corner” behavior accompanied by unresponsiveness to the attacker’s movements or manipulation of the cage by the experimenter (20). Since then, it has been demonstrated that IBA levels are greater in comparatively non-enriched (standard) home-cage environments versus larger, highly enriched preferred cages (21–25). Furthermore, greater time spent IBA in the home cage tends to predict greater time spent immobile in the forced swim test [Fureix et al. (21), see in MacLellan et al. (18) for replication], and has been associated with three other symptoms of human clinical depression: anhedonia (assessed by reduced preference for sucrose, Trevarthen et al., in prep) and changes in weight and sleep [MacLellan et al. (18), replicated in Trevarthen et al., in prep]. Moreover, chronic administration of Venlafaxine (a serotonin-norepinephrine reuptake inhibitor antidepressant), exposure to housing conditions promoting better health and well-being, and their combinations alleviate IBA levels (24), therefore showing common curative factors between IBA in mice and human clinical depression. Altogether, the results suggest a depressive-like state in laboratory mice displaying greater levels of IBA in their home-cage. Here we explored this hypothesis further by investigating whether combining genetic predispositions and/or aversive early life experiences to environmental stress in adulthood triggers further IBA in mice, just as it can do in humans.

We tested genetic predispositions by using two common laboratory strains of mice, C57BL/6 J and DBA/2 J, known to differ in their tendencies to display depression-like profiles under tests, with C57BL/6Js being more prone to display depression-like profiles [e.g., (26, 27)] although phenotypes may vary between laboratories [e.g., (28)]. These strains also differ in their IBA levels, and even more so when housed in relatively barren ‘shoebox’ cages, although the direction of the difference can vary between studies. Indeed, C57BL/6 s have been shown to display more IBA than DBA/2Js in Fureix et al. (21) and Nip et al. (23) (Canadian lab, mice sourced from US Charles River), but DBA/2Js displayed more IBA than C57BL/6 s in Trevarthen et al. (25) (British lab, mice sourced from European Charles River), and strains did not differ in Fureix et al. (24). That behavioral strain phenotype may be sensitive to supplier and/or unidentified ‘local environment’ effects is not uncommon [e.g., (28–30)].

To manipulate the early life experience of mice, maternal separation was applied pre-weaning to half of the mice for each strain (other half undisturbed: control group). Maternal separation is a form of early life stress, which is designed to simulate parental neglect by separating dams and pups for an amount of time (variable between studies). Applying maternal separation to induce depressive features in animals shares construct validity with the human illness (31), since people exposed to early life stressors are at higher risk of developing depression, e.g., (32, 33). Moreover, maternal separation results in long-lasting behavioral and neuroendocrine alterations in animals similar to behavioral and neuroendocrine features that can be observed in depressed people [face and construct validity, (31)]. For instance, female CD1 mice submitted to maternal separation showed an imbalance in the tryptophan-kynurenine pathway in brain areas associated with emotional processes (pre-frontal cortex and hypothalamus) (34). In humans, major depression is associated with increased inflammatory drive, which causes alterations in the tryptophan-kynurenine pathway and thus the production of neurotransmitters regulating mood such as serotonin (35). Female CD1 mice submitted to maternal separation also show greater depressive-like behavior in tail suspension (greater amount of time spent immobile) and saccharin preference (lower preference at 24 h) tests (34), respectively used as proxies for two symptoms of depression in humans [hopelessness and anhedonia (2)]. Maternal separation in mice also shares predictive validity with human depression since treatment with fluoxetine attenuates the effects of maternal separation (36, 37). Finally, variations in environmental stress at adulthood were induced by housing the mice post-weaning in either barren ‘shoebox’ cages [i.e., validated as inducing depression-like features (38) and greater levels of IBA (21, 22) in rodents], or larger, highly enriched preferred cages.

Our first prediction is primarily methodological: that maternal separation would reduce the maternal behavior of the dams (assessed here using time spent active in the nest as a proxy), therefore confirming a differential early life condition treatment for the pups, which would subsequently influence their susceptibility to depression-like states in adulthood. Since chronic stress commonly triggers depression in humans, particularly in people with genetic predisposition and/or exposure to early life stress, our second prediction is that relatively barren, non-preferred small cages (‘non-enriched’) would trigger greater levels of IBA than larger, highly enriched preferred cages, and even more so in mice exposed to early life maternal separation stress and/or one of the two strains (39, 40). Due to the variation in strain differences between above mentioned study, we predict a strain-related difference exacerbated in non-enriched cages, rather than one strain specifically displaying more IBA than the other.

## 2 Materials and methods

### 2.1 Ethical approval

**Figure 1:**
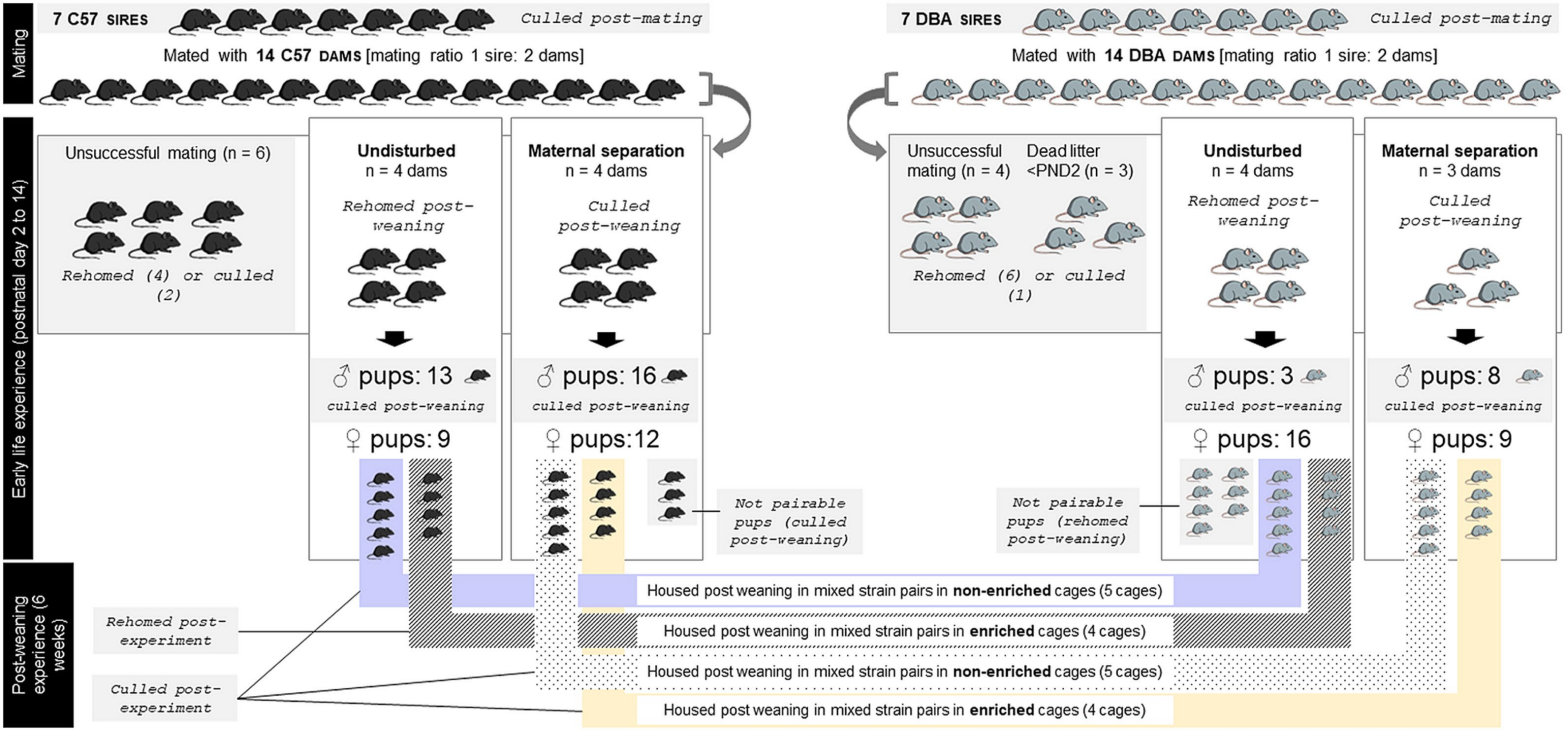
Overview of the experimental design, and number and fate of mice used at every step of the experiment. **C57** = C57BL/6 J mice, **DBA** = DBA/2 J mice.

This study was conducted under the UK Home Office Licences PPL P2556FBFE and P10DC2972, in accordance with the Animals (Scientific Procedures) Act 1986 (ASPA), the EU directive 2010/63/EU and the UK Home Office code of practice for the housing and care of animals bred, supplied, or used for scientific purposes. The mice were handled using a polycarbonate tunnel from their home cage, following a validated method shown to reduce stress in laboratory mice (41), at all times unless stated otherwise. Animals were monitored daily throughout the study for any health issues; none were observed at any point. At the end of the study, the mice were either euthanized by skilled technicians (using concussion, immediately followed by cervical dislocation and confirmation of death; the use of both methods ensured rapid loss of consciousness), or released from the ASPA and rehomed to private owners subject to the conditions stated in the PPL P2556FBFE (n _rehomed_ = 33 out of 128 mice used) (Figure 1).

### 2.2 Animals and overview of experimental treatments

This experiment was conducted at the research facilities of the University of Bristol from January to March 2020. Estimates of animal numbers were based on power analyses using data from Trevarthen et al. (in prep) (power 80%, significance criterion 2-tailed 0.05, Cohen’s d effect size 0.7 (minimum expected means difference 0.011 and estimated standard deviation 0.016), replicated using several calculators). The Covid-19 pandemic situation, together with funding completion at the time the facilities re-opened post-pandemic, however prevented us from completing the experiment on the last batch of mice. In total, 71 C57BL/6Js (‘C57’) and 57 DBA/2Js (“DBA”) were used: 14 dams and 7 sires of each strain (Charles River, France, eight weeks old at arrival), and their 50 C57 and 36 DBA offspring (bred at our facility). At birth, dams of each strain and their pups were randomly split between two treatments: either maternal separation or left undisturbed (Figure 1). At weaning, the pups were pseudo-randomly (matching cage mates for weight) assigned into mixed strain pairs randomly split between two different environment conditions: either in standard ‘shoebox’, relatively barren cages, or in larger highly enriched cages (Figure 1). Only female pups that could be paired by ELS condition at weaning (*n* = 18 of each strain) were exposed to the post-weaning differential environment condition. Indeed, depression is more prevalent in women (3) (although we would predict greater level of IBA to reflect depression-like states in males as well), and our mixed-strain housing promoting Refinement (see below) has been investigated and validated in females, not in males (42, 43). The remaining 10 female offspring which could not be paired at weaning were either culled following Schedule 1 killing method (*n* = 3) or rehomed (*n* = 7). All the male pups (*n* = 40) were culled following Schedule 1 killing method, out of which 16 were first released from The Act, kept alive at the licensed establishment and used in non-procedural (reward acquisition based) behavioral work piloting for a different study.

### 2.3 Breeding conditions

Male breeders were housed in randomly allocated single-strain groups of three to four individuals, and females in single-strain pairs. Each cage had its own ventilation system regulated by an airflow system (IVC cages, 30 cm L × 20 cm W × 17 cm H; Model EFS120PR00; Allentown Inc., New Jersey, United States) and contained nesting material, a cardboard igloo and a Perspex tunnel. All cages were housed within a single rack in the same colony room (room temperature: 21°C, humidity level: 37–45%). The light–dark cycle was 12/12 h with lights on 0800–2000. Two weeks after arrival, a single male (randomly chosen) was introduced in a females’ home cage for 7 days, allowing both females to mate. The onset of gestation was determined by daily observation of the females’ genital area (presence of a vaginal plug) conducted by experienced animal care staff. Females remained housed in pairs until 5–8 days before parturition date, then they were singly housed in the IVC cages above described to allow better parturition and maternal care [cf. (44)].

### 2.4 Early life experience treatment

Litters were equally split between either the maternal separation or the undisturbed (control) treatment, balancing assignment for each of birth date (post-natal day 0; PND0), litter size (number of pups born alive) and sire (paternal origin) following a randomization schedule (Figure 1). The housing of the cages within the rack was reorganized in a pseudo-random order that ensured an equal spreading of treatments (strain and early life experience) within shelves. Maternal separation happened daily for 180 min from post-natal day (PND) 2 to 14 (Figure 1). Applying 180 min of daily maternal separation has been shown in the literature to induce neuro-physiological (e.g., decreased Brain-Derived Neurotrophic Factor mRNA in the hippocampus, increased 5-HTT methylation) and behavioral (e.g., forced swim test, sucrose consumption) changes reflecting depressive phenotypes in the pups post-weaning (45). The time of the day at which the litters underwent the maternal separation was randomized (Table 1) to increase unpredictability of the event. During maternal separation, the home cages were removed from the rack and placed on heat pads (temperature controlled at 21°C) for 3 h, during which the dams were removed from their home cage and placed alone in another IVC cage back into the rack, in the same room as the pups. Each dam had her own ‘separation cage’ to avoid extra stress due to mixing smells, with fresh bedding added in the dam separation cage on the first separation day and not changed afterwards. Food and water were provided but no enrichment was added. Control litters were checked daily for the pups’ and dams’ health condition, but no disturbance was made to the nest besides lifting the house/food hopper. For both treatment groups, the pups remained undisturbed until they were sexed (PND 20), and the cages were not cleaned until weaning, unless necessary (*i.e* flooding, one cage).

**Table 1:** Maternal separation treatment timetable.

Post-natal day	Maternal separation scheduled on:
2	1130-1430
3	0900-1200
4	1400-1700
5	1200-1500
6	1130-1430
7	1000-1300
8	1300-1600
9	1200-1500
10	1130-1430
11	0930-1230
12	1400-1700
13	1230-1530
14	1130-1430

### 2.5 Post-weaning differential environment condition

Offspring were weaned at 26.9 ± 2.90 day-old (range: 25–28 days-old). At weaning, the dams that did not undergo the maternal separation (*n* = 8 mice) and females for which mating was not successful (*n* = 10; C57: *n* = 6; DBA: *n* = 4) or which litters died before PND2 (*n* = 3; DBA: *n* = 3) were rehomed (*n* = 10) or culled (*n* = 3). The dams exposed to the maternal separation treatment (*n* = 7 mice) and sires (*n* = 14) were culled following Schedule 1 killing method. Offspring were moved at weaning to a different colony room (temperature: 21°C, humidity: 37–45%). Each mouse was weighed, and one C57 and one DBA female mouse of the same early life stress treatment and of similar weight (mean weight difference across all pairs = 1.0 g [range 0–3.7 g]) was pseudo-randomly allocated to each cage (mouse selected at random, but matched with cage mate based on weight). Mixed-strain pair-housing allows non-invasive identification of the individuals within pairs, removing the need to conduct invasive identification during behavioral observations (42). For each of the early life conditions, the cages were pseudo-randomly split (i.e., counterbalancing full- and half-siblings across post-weaning environment conditions) between two post-weaning environmental conditions: either in standard, relatively barren cages (“NE”, 10 cages), or in larger highly enriched cages (‘EE’, 8 cages)^2^ (Figure 1) in which they were housed for 6-weeks.

The NE cages were conventional laboratory cages (37 cm L × 21 cm W × 14 cm, Techniplast) equipped with basic enrichments: sawdust (IPS), a handful of nesting material (Datesand Bed-R’Nest), a Polycarbonate handling tunnel (13 cm L, ∅ 5 cm, Datesand) and a small piece of cardboard. The EE cages were larger (44 cm L × 34 cm W × 20 cm H; Techniplast), and equipped with sawdust, a larger amount (three handfuls) of nesting material (Datesand Bed-R’Nest), the polycarbonate handling tunnel, two small pieces of cardboard, a running wheel with a red igloo (fast-trac, Datesand), one plastic transparent shelter (∅ 15 cm, 5.5 cm H, Biopac UK), three wooden blocks (two small: 5 cm L × 1 cm W × 1 cm H, one large: 10 cm L × 2 cm W × 2 cm H, Datesand), a pinecone (autoclaved before being placed in the cage), additional nesting material such as tissue (Waitrose Basics), two Nestlets (Ancare, United States), two Cocoon nestlets (Datesand) and Sizzle Nest (Datesand), as well as (attached to the cage lid using cable ties): one flexible plastic tunnel (Merlett Superflex L Hose: 30 cm L × ∅ 6.3 cm, RS Components), one half of a coconut shell (approximate dimensions: 12.7 cm L × 7.6 cm W × 5 cm H, Little Cherry Ltd), a hammock made from pillowcases (approximately 15 cm × 8 cm), a sisal rope ladder, and a single spray of Millet (Pets at Home).

All cages were housed within two scantainers (*Scanbur,* Karlslunde, Denmark). Cages ID and location within the scantainers were allocated by an assistant (naïve to the study and not involved in behavioral observations later), in a pseudo-random order that ensured an equal spreading of experimental treatments (early life experience and post-weaning environment conditions) between the two scantainers, and within shelves for each scantainer. This ensured behavioral observers remained blind to the early-life treatment each cage of mice had been exposed to. NE cages were cleaned every week and larger EE cages were cleaned every three weeks. During cleaning, mice from NE cages were tail-handled [as part of the stress at adulthood treatment, see, e.g., (41)], while EE mice were tunnel-handled. Food (LabDiet) and water were available *ad-libitum* for all, and all mice were kept under a 12 h reversed light–dark cycle (lights on 1900–0700) to facilitate post-weaning in-cage behavioral observations. At the end of the experiment, the mice that did not undergo the maternal separation and were housed in an enriched environment (*n* = 8) were rehomed and the mice in the other experimental conditions (*n* = 28) were culled following Schedule 1 killing method.

### 2.6 Measures

Live behavioral observations (following habituation to the presence of the experimenter in the room) were conducted by two trained experimenters (OS and EF, Cohen’s Kappa overall interrater agreement during training = 94.45%, ranging from 87 to 97% according to the behavior considered, splitting equally the sessions between the two observers). Observation sessions took place both during the early life experience (to quantify the time dams spent active in their nest), and during the post-weaning differential environment condition phases (to quantify the behavior related to hypothesis under test, i.e., IBA). Inter-observer reliability was assessed during training of live observations sessions, where the two observers independently observed the same mice at the same time, and later compared their ratings. There were 4 training sessions (of 1 h each) in total. Two sessions were performed on non-experimental litters (i.e., not part of this study) to assess inter-observer reliability with regards to quantifying the dams’ maternal behavior. Two other training sessions were conducted post-weaning on the mice included in the experiment (data not included in the current study analyses), before the actual observation period started.

#### 2.6.1 Behavioral observations conducted during the early life experience phase

On PND2 (first day of maternal separation protocol), PND6, PND10 and PND14 (last day of maternal separation protocol), the time dams spent active in their nest (proxy for maternal behavior) was observed live every 5 min for 1 h before separation and 1 h following reunion, using instantaneous scan sampling method (12 scans/mouse/observation) (46). Control (undisturbed) mice were simultaneously observed to allow comparison of the activity in the nest between the two treatments. Mice were scored as ‘Active in the nest’ when grooming pups, moving nesting material, nursing pups, or performing rapid movements inside the nest [adapted from Brajon et al. (44)]. The dams and their litter were not yet allocated to their future post-weaning environment conditions, which ensured that the observers recording the dams’ behavior were blinded to this aspect of their treatment.

#### 2.6.2 Behavioral observations conducted during the post-weaning differential environment condition phase

The behavior relevant to the hypothesis under test (IBA) was defined following (23, 24) as ‘mouse motionless, muzzle in sight and eyes open, for at least 3 s’. Live behavioral observations of the mice in their home cage began after four days of acclimatization to the new colony room. All observations were conducted during the dark (active) phase under red ambient light, for six weeks (PND 30–33 to PND 67–70), two days per week (Tuesdays and Thursdays), over two 90-min time blocks per day (0930–1,100 and 1,100–1,230). The sampling timetable was chosen after analyzing data from previous experiments, indicating that the proportion of IBA observed weekly when sampling as described above predicted the proportion of IBA observed weekly when sampling more extensively on 4 daily blocks, 4 days per week (assessed and replicated independently across two laboratories; Trevarthen et al., MacLellan et al., unpublished data). Behavior was recorded via live instantaneous scan-sampling (46), switching from scan to 3 s focal sampling to allow for differentiation between behaviors characterized by a lack of movement (e.g., IBA versus sleeping) as in Fureix et al. (21). In total, 8 scan samples were taken per mouse each time block (i.e., scan performed every 10 min), totaling 32 scan samples/mouse/week, and 192 scan samples/mouse in total. The observers (EF and OS) were blind to the mice’s early life experiences, since they were not involved in allocating the post-weaning cages ID and location within the scantainers (as described in 2.5. Post-weaning differential environment conditions section).

### 2.7 Statistical analyses

Statistical analyses were performed using R software [version 3.6.1 with RStudio 1.2.1578; (47)], using the mice as experimental units and generalized linear mixed models. Blinding to treatments during the analysis was done by *CF* assigning new (temporary) codes to the experimental conditions in data files before the analysis (experimental conditions were ‘de-blinded’ to the other investigators post-analyses). The normality of data was checked by Shapiro–Wilk tests, and the analyses were performed using lme4 (48) and lmerTest (49) packages. Least square means for post-hoc pairwise comparisons were extracted using the lsmeans package (50).

In order to evaluate the effect of the maternal separation on the amount of maternal care received by the pups, the proportion of time spent active in the nest (proxy for maternal behavior) was analyzed using generalized linear mixed models, with pups’ age (i.e., PND2, PND6, PND10 and PND14), time (i.e., before or after separation), ELS (i.e., maternal separation or undisturbed), strain (i.e., DBA or C57), and their interactions as fixed effects, and cage as a random effect. The significance of explanatory variables and their interactions was determined via stepwise reduction of the model, using Log-likelihood ratio tests (LRTs), starting from the most complex model with all possible pairwise interactions to the simplest one that explained best the response variables, using a significance level of 0.05. The final model only included post-natal day as a fixed factor and the cage as a random effect. All models ran without convergence issue. The effect size of the full model was 0.42, with an associated analysis power of 0.3. The final model explained more variance than a null model, which included only the random effects (*p* < 0.05).

To explore whether early life stress and/or genetic predispositions triggers further IBA in mice exposed to post-weaning environmental stress, the proportion of IBA whilst in view during scan sampling performed by each individual mouse (averaged across a week for each of the six weeks of observation) was used as the dependent variable. The initial model included week (i.e., 1 to 6), strain (i.e., DBA and C57), ELS (i.e., maternal separation or undisturbed) and post-weaning environment (i.e., enriched EE or non-enriched NE cages), and their interactions as fixed effects, and mouse ID as a random effect. The final model included week, environment and their interaction as fixed effects, and mouse as a random effect. All models ran without convergence issue. The effect size of the final model was 0.6, with an associated analysis power of 0.55. The final model explained more variance than a null model, which included only the random effects (*p* < 0.0001).

Time spent active in nest and time spent IBA during scan sampling reported in the text are Least Square Means (LSM) ± standard error (SE).

## 3 Results

### 3.1 Maternal behavior (pre-weaning observations)

**Figure 2:**
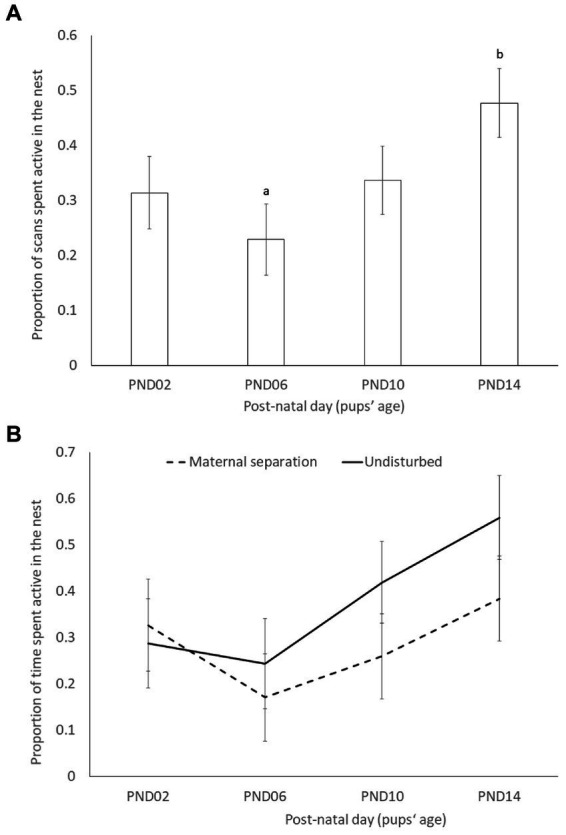
Least square means and standard errors of the proportion of scans spent active in the nest across pups’ age (PND = post-natal day; PND0 = birth and PND02 = start of maternal separation). **(A)** Pooled data (pups’ age effect: X^2^_3_ = 10.302; P = 0.016); **(B)** data split by early life stress treatment (pups’ age × ELS effect: X^2^_3_ = 4.3294; *p* = 0.228). Different superscript letters indicate significant differences between post-natal days at *p* < 0.05.

The proportion of scans the dams spent active in the nest significantly increased with pups’ age (X^2^_3_ = 10.302; *p* = 0.016), although only PND6 significantly differed from PND14 in post-hoc comparisons (t_91.9_ = −3.186; *p* = 0.0105; Figure 2). Contrary to the prediction, the time dams spent active in the nest was not significantly influenced by the early life stress treatment (maternal separation = 0.29 ± 0.061; undisturbed = 0.38 ± 0.059; X^2^_1_ = 1.6553; *p* = 0.2), time of observation (before/after separation) (before = 0.33 ± 0.051; after = 0.33 ± 0.051; X^2^_1_ = 0.047; *p* = 0.83), or by strain (C57BL/6 J = 0.28 ± 0.058; DBA/2 J = 0.38 ± 0.062; X^2^_1_ = 1.7641; *p* = 0.18). None of the 2-way, 3-way and 4-way interactions were significant (Table 2).

**Table 2:** Test statistics (Chi-Square), degrees of freedom (Df) and significance (*p*-value) of each fixed factor, and of the interaction between these factors, for the dams’ proportion of visible scans spent “active in nest”.

Model	Factor removed	Chisq	Df	*p*-value
ms1	None			
ms2	ELS*strain*age*time	2.1504	3	0.5418
ms3	Time*ELS*strain	1.0033	1	0.3165
ms4	Age*ELS*strain	4.5719	3	0.206
ms5	Age*time*strain	0.8649	3	0.8339
ms6	Age*time*ELS	0.122	3	0.9891
ms7	ELS*strain	0.9378	1	0.3328
ms8	Time*strain	0.0833	1	0.7729
ms9	Time*ELS	2.5936	1	0.1073
ms10	Age*strain	4.7141	3	0.194
ms11	Age*ELS	4.3294	3	0.228
ms12	Age*time	1.1508	3	0.7648
ms13	Time	0.047	1	0.8284
ms14	Strain	1.7641	1	0.1841
ms15	ELS	1.6553	1	0.1982
ms16	Age	10.302	3	0.01616

As an iterative approach was used for model selection, “Model” refers to the statistical model used for each step of the analyses, non-significant factors were removed in a step-wise way to obtain the final model. *p*-values were obtained by comparing each model to the previous one (i.e., the test statistics reflect the significance of the removed factor), and the final model (“ms16”) is highlighted.

### 3.2 Inactive but awake behavior (post-weaning observations)

**Figure 3:**
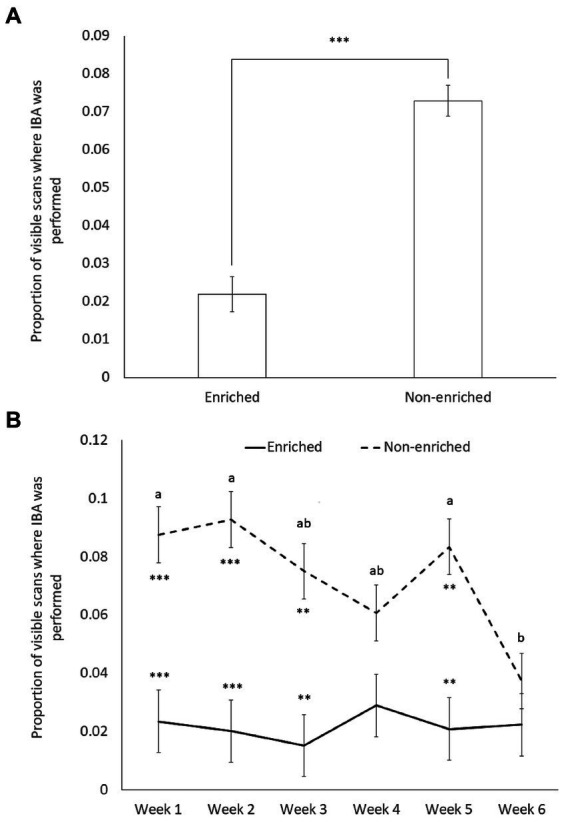
Least square means and standard errors of the proportion of visible scans displaying “inactive but awake” (IBA) behavior by mice housed in different post-weaning environments (enriched environment, *n* = 16; Non-enriched environment, *n* = 20). **(A)** Data averaged over the 6 weeks of experimentation (X^2^_1_ = 39.615; *p* < 0.0001); **(B)** data by week (week × environment: X^2^_5_ = 12.68; *p* = 0.027). Superscript letters indicate significant week differences within each environment at *p* < 0.05; environment differences are represented by ** for significance at *p* < 0.01 and *** for significance at *p* < 0.001.

As predicted, the proportion of visible scans displaying IBA across the 6 weeks was significantly lower in the mice housed in EE cages than in those housed in NE cages (X^2^_1_ = 39.615; *p* < 0.0001) (Figure 3A). Neither the early life experience (undisturbed: 0.045 ± 0.0045; maternal separation: 0.050 ± 0.0045) or strain (DBA: 0.048 ± 0.0045; C57: 0.047 ± 0.0045) significantly predicted the average proportion of visible scans displaying IBA (respectively X^2^_1_ = 0.6883, *p* = 0.4 and X^2^_1_ = 0.0404, *p* = 0.84). Contrary to the prediction, we also found non-significant 2-way and 3-way interactions (Table 3), i.e., no significant strain or maternal separation effects were observed when the mice were specifically housed in the NE (non-preferred), comparatively more stressful cages.

**Table 3:** Test statistics (Chi-Square), degrees of freedom (Df) and significance (*p*-value) of each fixed factor, and of the interaction between these factors, on the mice’ proportion of visible scans displaying IBA (averaged across the 6 weeks post-weaning).

Model	Factor removed	Chi-square	Df	*p*-value
m1	None			
m2	ELS*strain*environment*week	6.9257	5	0.2262
m3	Week*strain*environment	3.2928	5	0.6549
m4	Week*ELS*environment	6.059	5	0.3005
m5	Week*strain*ELS	4.881	5	0.4306
m6	Strain*ELS*environment	1.0576	1	0.3038
m7	Week*strain	2.1961	5	0.8214
m8	ELS*week	6.5641	5	0.2551
m9	Strain*environment	0.3204	1	0.5713
m10	ELS*strain	1.2496	1	0.2636
m11	ELS*environment	1.1379	1	0.2861
m12	ELS	0.6883	1	0.4067
m13	Strain	0.0404	1	0.8407
m14	Week*environment	12.68	5	0.02657
m15	Week	11.675	5	0.03953
m16	Environment	39.615	1	3.09E-10

As an iterative approach was used for model selection, “Model” refers to the statistical model used for the analysis, non-significant factors were removed in a step-wise way to obtain the final model. *p*-values were obtained by comparing each model to the previous one (i.e., the test statistics reflect the significance of the removed factor), and the final model (“m14”) is highlighted.

Finally, although no specific predictions were made about the week factor, week (X^2^_5_ = 11.675; *p* = 0.040) and its interaction with environment (X^2^_5_ = 12.68; *p* = 0.027) significantly predicted the proportion of visible scans in which mice performed IBA (Table 3). Hence levels of IBA were significantly lower in enriched than in non-enriched mice for most observation periods (week 1: t_204_ = −4.45, *p* = 0.0008; week 2: t_204_ = −5.04, *p* = 0.0001; week 3: t_204_ = −4.16, *p* = 0.0027; week 5: t_204_ = −4.38, *p* = 0.0013), except during week 4 (t_204_ = −2.211, *p* = 0.5435) and week 6 (t_204_ = −1.04, *p* = 0.9966). During week 6 the levels of IBA in NE mice dropped significantly compared to week 5 (week 5 vs. week 6: t_170_ = −3.44, *p* = 0.0345) (Figure 3B).

## 4 Discussion

The present study investigated whether early life stress and/or genetic predispositions (i.e., two risk factors increasing vulnerability to depression in humans exposed to stressful life events) also trigger greater time spent IBA in mice exposed to stress at adulthood. As predicted, barren post-weaning environment significantly enhanced IBA levels, which confirmed previous findings, e.g., (21–23). However, exposure to early-life stress via maternal separation did not significantly influence the likelihood of displaying IBA when exposed to a barren post-weaning environment. Furthermore, although the studied strains of mice (i.e., C57/6 J and DBA/2 J) significantly differed in their likelihood of displaying IBA in other studies (21, 23, 25), the present study did not find such an effect of genetic predisposition, as in Fureix et al. (24). Therefore, the results provide only partial support to our hypothesis that greater levels of IBA in mice are triggered by similar risk factors to those causing depression in humans. Several reasons could explain the lack of significant effects of maternal separation and strain on the level of IBA expressed in barren environments, which we discuss below.

First, this study started January 2020 and had to be interrupted due to the Covid-19 pandemic and could not be resumed after interruption (due to grant ending). Consequently, the sample sizes are lower than those originally planned from the power analysis, reducing the statistical power. Despite our reduction in statistical power, we still observed the differential housing treatment effect, which here was robust enough to reach significance regardless of the lack of power. That non-enriched cages trigger greater levels of IBA has indeed been replicated both across and within research institutes [Guelph, Canada: (21, 23); Bristol, UK: (24, 25)]. Such impoverished housing is also known to drastically impact mice welfare [e.g., increased stereotypic behaviors and aggression, weakened immunity, poorer breeding performances, and shortened life spans (22, 51–55)], and its detrimental effect might be too strong to be protected by the undisturbed early life condition. In contrast, the effects on IBA/ depressive-like phenotypes of genetic predispositions and exposure to early life stress might be less strong (as can be the effect of early life stress and genetic factors on human clinical depression) [e.g., (5, 8, 56) as discussed below]. Therefore, their statistical investigation may have been more likely to be jeopardized by the decreased statistical power. This study must therefore be replicated with originally planned (larger) sample sizes (i.e., 11 mice per strain × ELS × enrichment condition, i.e., 88 individuals in total).

With regards to genetic factors, human depression is only moderately heritable (~40%), and a highly polygenic disease, i.e., influenced by very high numbers of gene variants of very small effects, rather than being triggered by a few gene variants of large effects [e.g., (5–7)]. While genetic animal models of depression such as knockout mice (generated by single gene deletion) have provided important information about the exact role of specific genes in the pathophysiology of the illness, they have also shown limited applicability to the complex processes that occur within human depression, since they do not encompass the highly polygenic feature of the illness, e.g., (57). Therefore, while genetic factors linked to neuronal growth, synaptic function and inflammation are at stake in triggering susceptibility to clinical depression, the exact genetic risk factors predisposing some people to the illness are not yet fully understood, e.g., (6, 7). Moving away from testing the effect of specific gene(s) and conducting genome-wide association studies (a hypothesis free or ‘unbiased’ approach to detecting gene variants involved in an illness) is recognized as a way to move forward for animal models and human investigation. Such approaches are however not free from difficulties [e.g., very large sample sizes are required to have sufficient power to detect effects at a statistical threshold, as discussed in detail in, e.g., (8)]. Moving towards interdisciplinary ethological x genome-wide association studies could nevertheless be a way to take the IBA investigation further.

The effect of the second predisposing risk factor to clinical depression we investigated here, i.e., exposure to early life stress, can be sensitive to the research design [e.g., (5, 58)]. For instance, in humans, variations in defining and measuring early life stressors [e.g., (32, 59, 60)] and in methods used to assess the depressive outcomes [e.g., (5, 7, 8)] can result in different findings between studies. In animal models investigating how maternal separation impacts the development of depressive-like phenotypes, variations in the maternal separation procedure (61–63), housing and caring procedures, early life experience of the dam, sex of the pups and sample sizes can also influence the results (64). Creating a single maternal separation paradigm that would be fully identical and replicable across laboratories and within studies seems (realistically) unachievable. Combining results from investigations, each using different early life stressors (e.g., reduced bedding and nesting material (64), early weaning) may be an approach to target this replication difficulty. Alternatively, a potentially Refined approach with regards to the 3Rs (Mason, personal communication) to study the effect of early life stress on IBA development could be to create a contrast between early life conditions by making the control treatment much more positive than what it is normally (e.g., house the dams in highly enriched cages, allow natural weaning from the dams), rather than by making the early life environment more negative to create the early life stress. Studying the effect of maternal separation as an early life stressor nevertheless remain relevant for the study of welfare of captive species [especially dairy, e.g., (65, 66)], which are often weaned much earlier than they would be under natural conditions (67).

Any one (or all) of the explanations discussed above may be responsible for our partially supported hypothesis that greater levels of IBA are triggered in mice by similar risk factors to those causing depression in humans. We can also question whether the failure to induce a significant difference in actual maternal attendance at the nest/offspring may also contribute to the lack of effect of maternal separation observed in our study, as it was suggested that the amount of maternal care would influence the emotional response of offspring, independently of the maternal separation treatment (68). Furthermore, maternal separation was conducted in the colony room for practical reason (space availability). We therefore cannot rule out the possibility that mice from the control undisturbed group may have been affected by maternal separation by being exposed to the treatment groups’ vocalizations. A further replicating study would need the maternal separation treatment to be applied in a different room from the room housing the undisturbed mice. Another possibility could be that genetic background and early life stress do not increase IBA levels when exposed to stress later in life because elevated IBA does not reflect a depression-like state. We must for instance cautiously acknowledge the possibility that greater levels of IBA in the home environment can be associated with a different affective state, such as tentatively putative boredom-like states in dogs (*Canis lupus familiaris*) (69). Greater levels of IBA in mice nevertheless covaries with other symptoms of depression [i.e., immobility in the forced swim test (21, 23), changes in sleep and weight (18), Trevarthen et al. in prep] and are reduced by administration of antidepressants (24), making testing further the depression-like state hypothesis worthwhile. Therefore, future research replicating the current study should be conducted using larger sample sizes and using several paradigms of early life stress. Furthermore, co-existence and co-variation of greater levels of IBA with the range of the human illness symptoms that can be operationalized in animals should be investigated, since co-existence of symptoms is of crucial relevance to the diagnosis of the condition in humans (18, 70).

## 5 Conclusion

This study further supports that barren ‘shoebox’ cages trigger a specific form of waking inactivity (IBA) in laboratory mice. Although no significant effect of early-life stress and genetic factors could be evidenced in this study, we encourage further research on this topic, especially studies using larger sample sizes, several paradigms of early life stress, and investigating the co-existence and co-variation of greater levels of IBA with the range of human depression symptoms that can be operationalized in animals.

## Data availability statement

The datasets presented in this study can be found in online repositories. The names of the repository/repositories and accession number(s) can be found at: https://osf.io/94bf6/?view_only=b2dfebf15c5e470bbb8a85c5921c83f7.

## Ethics statement

The animal study was approved by Home Office User Research Ethics Board (Licence number PPL P2556FBFE and P10DC2972). The study was conducted in accordance with the local legislation and institutional requirements (Animals Scientific Procedures Act 1986 (ASPA), the EU directive 2010/63/EU and the UK Home Office code of practice for the housing and care of animals bred, supplied, or used for scientific purposes).

## Author contributions

OS: Data curation, Formal analysis, Investigation, Methodology, Visualization, Writing – original draft, Writing – review & editing. EF: Investigation, Methodology, Writing – review & editing. AT: Formal analysis, Methodology, Project administration, Supervision, Writing – review & editing. CW: Formal analysis, Writing – review & editing. EP: Methodology, Writing – review & editing. MM: Conceptualization, Funding acquisition, Project administration, Supervision, Writing – review & editing. CF: Conceptualization, Data curation, Funding acquisition, Methodology, Project administration, Supervision, Writing – review & editing.

## Footnotes

^1^A term by which we mean “major depressive disorder” or having “depressive episodes”, to encompass Diagnostic Manual of Mental Disorders 5th edition & International Statistical Classification of Diseases & Related Health Problems ICD-11 terminologies.

^2^At weaning, we obtained 9 pairs of mice (one C57 + one DBA) for each ELS condition (MS and UND), of which 4 pairs were assigned to “NE” and 5 were assigned to “EE.”
